# The Chimney/Periscope Technique as Total Endovascular Treatment of Kommerell's Diverticulum

**DOI:** 10.1055/s-0041-1729850

**Published:** 2021-10-29

**Authors:** Marco Zavatta, Francesco Squizzato, Alberto Dall'Antonia, Michele Piazza, Michele Antonello

**Affiliations:** 1 Department of Cardiac, Thoracic and Vascular Sciences, Vascular and Endovascular Surgery Clinic, Padova University, School of Medicine, Padova, Italy

**Keywords:** Kommerell's diverticulum, TEVAR, chimney, periscope

## Abstract

We report a case of Kommerell's diverticulum (KD) treated with a total endovascular approach, maintaining supra-aortic trunk (SAT) patency. A 75 year-old female with aneurysmal KD was deemed unsuitable for open surgery. Landing zone 2 was unfeasible; therefore, we planned an endovascular approach with landing in zone 1, chimney to left subclavian artery and periscope to right subclavian artery. Postoperatively she was free from complications, with complete exclusion of KD and SAT patency at 3-year follow-up.

## Introduction

Kommerell's diverticulum (KD) is a dilatation of the origin of an aberrant artery arising from the descending aorta. This congenital anomaly may predispose to aneurysmal evolution, dissection, rupture, and compression of adjacent structures. Surgical repair is advocated in symptomatic patients or in cases of KD diameter >30 mm and distance to the opposite aortic wall (DAW) >50 mm. 1 2 3 The treatment may consist of open surgical, hybrid, or total endovascular approach.

We report a case of KD in the setting of a left-sided aortic arch successfully treated with a total endovascular approach under local anesthesia. Chimney technique was used to allow a safe landing in zone 1 and periscope technique was used to maintain patency of the aberrant right subclavian artery (ARSA) in a retrograde fashion.

## Case Presentation

**Fig. 1:**
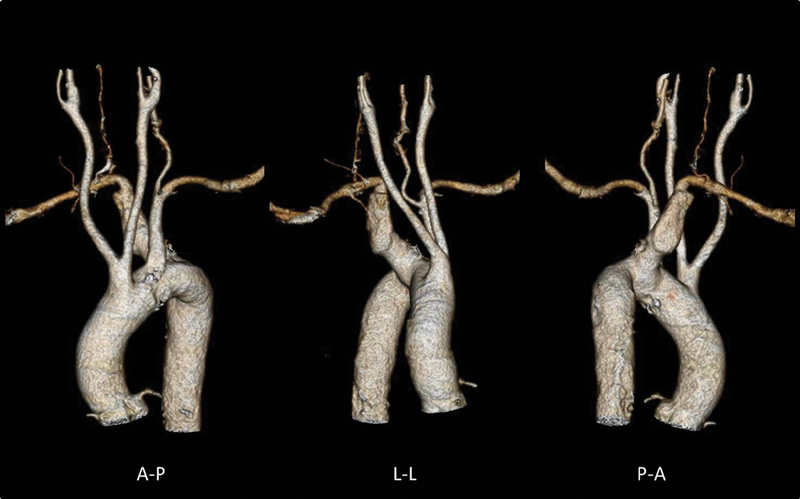
Preoperative angio–computed tomography three-dimensional reconstruction. A-P, anterior-posterior; L-L, lateral-lateral; P-A, posterior-anterior.

The technique is demonstrated in a 75-year-old female with severe chronic obstructive pulmonary disease. An angio-computed tomography (CT) scan demonstrated the presence of an aneurysmal KD of a right aberrant subclavian artery with maximum diameter of 51 mm and a diameter of 32 mm at its origin, in the setting of a left-sided aortic arch. Left and right common carotid arteries originated from a common trunk ( Fig. 1 ).

The case was discussed with the anesthesiology team and deemed unsuitable for open surgery and general anesthesia. Therefore, the patient was scheduled for a total endovascular repair under local anesthesia. Informed consent for case report has been acquired.

Landing in zone 2 was unfeasible due to anatomic reasons. The distance between ARSA and left subclavian artery (LSA) was only of 7 mm. Therefore, we had to plan the landing of a thoracic endograft in zone 1, with chimney technique to revascularize LSA and periscope technique to revascularize the ARSA. With this setting, the total length to be covered was 100 mm with a proximal diameter of 28 mm. Access was poor, with small and diseased iliac arteries. Therefore, to ensure a 20% aortic oversizing, a low profile Cook Zenith Alpha 34–113 (Cook Inc., Bloomington, IN) was chosen.

The procedure was performed in a surgical hybrid suite, under local anesthesia.

After performing a right femoral and a left brachial cut down for access, the patient was heparinized with an activated clotting time goal of 300 seconds. From the right femoral artery, a Lunderquist (Cook Medical, Bloomington, Indiana) stiff guide was placed into the ascending aorta, followed by a pigtailed catheter inserted from the left brachial access.

A Cook Zenith Alpha 34–113 (Cook Inc) was advanced into the aortic arch. Under road map angiography, we advanced and deployed a 10–8 covered stent (Fluency, Bard Inc., Karlsruhe, Germany) between the ascending aorta and the LSA, reinforced with a nitinol bare metal stent (Protégé GPS 10–6, Ev3, Plymouth, MN).

**Fig. 2:**
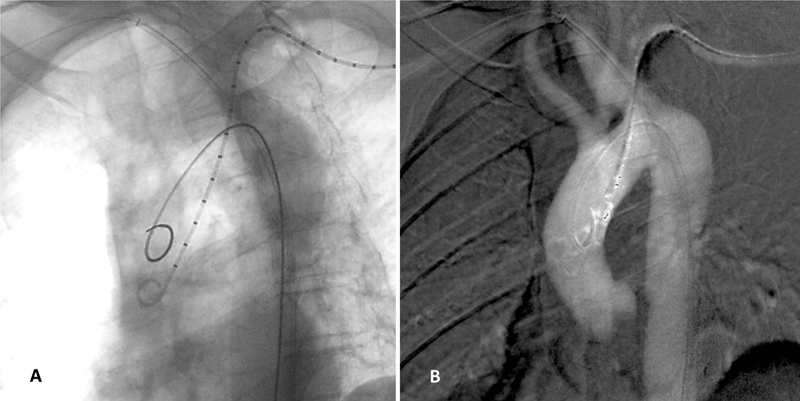
( **A** ) Fluoroscopic image and ( **B** ) road map angiogram showing intraoperative cannulation of aorta, aberrant right subclavian artery and left subclavian artery.

From the left femoral access, we introduced a stiff guide into the right axillary artery ( Fig. 2 ). Hereafter, we advanced and deployed, through a 7 Fr Destination guiding sheath, (Terumo, Japan) two Viabahn endografts (W.L. Gore and Associates, Flagstaff, AZ), 8–15 and 8–10, between the ARSA and the descending aorta. We reinforced also the Viabahn endografts with bare metal stents (Protégé GPS 8–60 and 8–40, Ev3).

We performed a second left transfemoral puncture and we advanced a 5 Fr VER (Cordis Corp, Bridgewater, New Jersey) catheter into the ascending aorta. Under road map angiography, we deployed the Zenith Alpha thoracic endograft just distally to the origin of the carotid common trunk. From the VER catheter, now positioned between the aortic wall and the endograft, we performed an intraluminal spiral embolization of the aneurysmal diverticulum.

At final intraoperative angiogram, the thoracic endograft appeared well positioned with patency of the carotid common trunk and of the LSA with antegrade flow from the chimney graft and of the ARSA with retrograde flow from the periscope. The KD appeared to be completely excluded.

The procedure was uneventful, and the overall procedure time was 240 minutes.

**Fig. 3:**
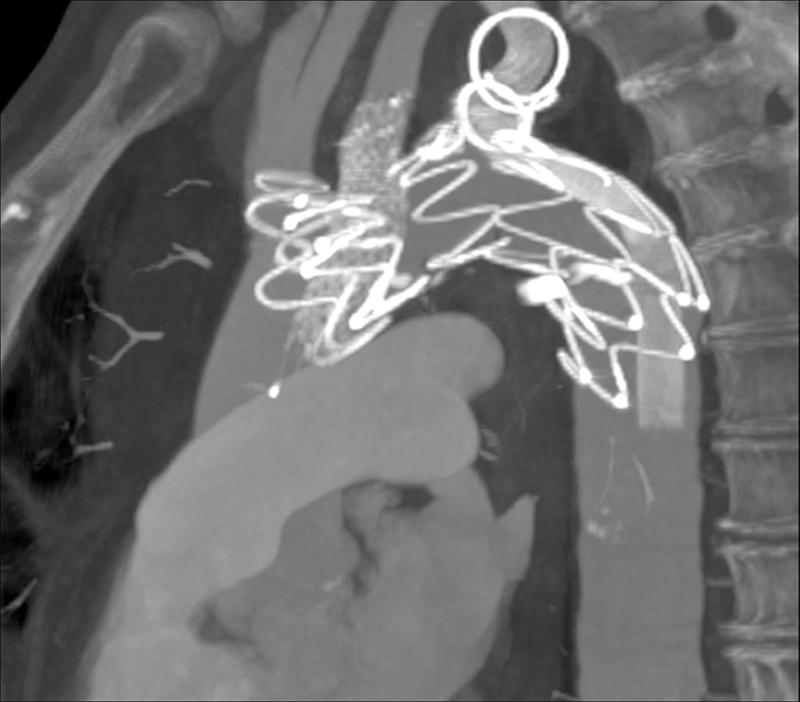
Three-year follow-up angio–computed tomography scan.

The patient was discharged after 3 days with no complications, under double antiplatelet therapy. Angio-CT scans performed at 3 months, 1 year, 2, and 3 years demonstrated complete exclusion of the KD and patency of the supra-aortic trunks (SATs; Fig. 3 ).

## Discussion

KD is a rare congenital malformation that was first described in 1936, caused by the persistence of a remnant of the right dorsal aorta located between the definitive descending aorta and the origin of the right subclavian artery. During adulthood, concomitant atherosclerotic degeneration may predispose to rupture or dissection. Preventive treatment is indicated in symptomatic cases or in case of a diverticulum size >3 cm, aberrant subclavian artery orifice diameter >3 cm, or DAW > 5 cm. 1 2 3

Open surgical treatment usually consists of aortic arch replacement and supra-aortic vessel revascularization via a median sternotomy. Technical difficulties may occur, especially regarding secure cerebral perfusion during surgery, if an ARSA is present. Nowadays, the adoption of an open surgery approach is justified only in patients with associated congenital abnormalities requiring concomitant correction or with good surgical risk.

An endovascular approach today represents a valid option. Cases of total endovascular treatment have been reported in literature. The majority of these consisted of TEVAR followed by embolization of the ARSA. 4 This technique has the advantage to be minimally invasive and the virtual benefit to reduce mortality and general complications. On the other hand, subclavian artery coverage without revascularization may cause arm claudication and predispose to spinal ischemia or vertebrobasilar insufficiency. These risks can be reduced by subclavian artery revascularization through an open surgical bypass. Hybrid treatment is indeed the most frequently used technique for KD repair in the last years. 5 6 Nevertheless, this still requires general anesthesia and does not eliminate risks related to open treatment.

A case of total endovascular repair of KD maintaining SAT patency has been reported by Silveira et al 7 in the setting of a right-sided aortic arch. They used a surgeon-modified thoracic endograft to create an internal branch attached in a retrograde fashion. Similarly, Gafoor et al 8 described the treatment of an ARSA aneurysm using a custom-made endograft.

We agree with these authors that total endovascular treatment maintaining SAT patency under local anesthesia should be considered as first-line treatment in high–surgical risk patients.

In this report, we propose another possible total endovascular solution for KD treatment with preservation of SAT patency. In the presented case, a proximal antegrade chimney for the LSA was necessary to achieve a safe sealing. ARSA patency was obtained through a retrograde approach for two reasons as follows: (1) to improve proximal sealing and reduce the risk of gutter endoleak, avoiding an excessive burden of stents in the proximal sealing zone; and (2) to reduce the angulation of the stent graft between the ARSA and the thoracic endograft. In fact, the choice to perform a retrograde revascularization was also related to the presence of a sharp angulation between the ARSA origin and the aortic arch, as the angle with descending aorta was more favorable.

The covered stents used were reinforced using bare metal stents to reduce the risk of stent kinking or compression by the endograft. At the same time a 20% oversizing of the thoracic endograft was planned to promote proper proximal sealing and reduced the risk of prosthesis migration.

To the authors' knowledge, few cases of total endovascular treatment of KD associated with SA revascularization have been reported, and this is the first case with the chimney/periscope technique. The advantages of this technique are related to the possibility to perform the procedure under local anesthesia, with off-the-shelf devices, reducing surgical and postoperative risks. This procedure represents a technically challenging endeavor and it is an off-label application.

## References

[1] Ota T Okada K Takanashi S Yamamoto S Okita Y Surgical treatment for Kommerell's diverticulum J Thorac Cardiovasc Surg 2006 131 03 574 578 16515907 10.1016/j.jtcvs.2005.10.012

[2] Erben Y Brownstein A J Velasquez C A Natural history and management of Kommerell's diverticulum in a single tertiary referral center J Vasc Surg 2020 71 06 2004 2011 31708305 10.1016/j.jvs.2019.08.260

[3] Tanaka A Milner R Ota T Kommerell's diverticulum in the current era: a comprehensive review Gen Thorac Cardiovasc Surg 2015 63 05 245 259 25636900 10.1007/s11748-015-0521-3

[4] Gao P Wang M Dong D Kong X Jin X Zhang S Endovascular repair of a Kommerell diverticulum anomaly Ann Thorac Surg 2015 99 05 1801 1803 25952212 10.1016/j.athoracsur.2014.06.094

[5] Frigatti P Grego F Deriu G P Lepidi S Hybrid endovascular treatment of aneurysm degeneration in a rare right-aortic arch anomaly with Kommerell diverticulum J Vasc Surg 2009 50 04 903 906 19576720 10.1016/j.jvs.2009.04.065

[6] van Bogerijen G H Patel H J Eliason J L Evolution in the management of aberrant subclavian arteries and related Kommerell diverticulum Ann Thorac Surg 2015 100 01 47 53 25912743 10.1016/j.athoracsur.2015.02.027

[7] Silveira P G Franklin R N Cunha J R Neves T T Nascimento G G Bortoluzzi C T Total endovascular repair of aberrant left subclavian artery with Kommerell's diverticulum using a customized branched device J Vasc Surg 2013 57 04 1123 1125 23312832 10.1016/j.jvs.2012.10.008

[8] Gafoor S Stelter W Bertog S Sievert H Fully percutaneous treatment of an aberrant right subclavian artery and thoracic aortic aneurysm Vasc Med 2013 18 03 139 144 23720037 10.1177/1358863X13485985

